# Disputing Lamarckian epigenetic inheritance in mammals

**DOI:** 10.1186/s13059-015-0626-0

**Published:** 2015-03-27

**Authors:** Emma Whitelaw

**Affiliations:** Department of Genetics, La Trobe University, Bundoora, VIC 3086 Australia

## Abstract

A recent study finds that changes to transcription and DNA methylation resulting from *in utero* exposure to environmental endocrine-disrupting chemicals are not inherited across generations.

## Epigenetic reprogramming

All mammals develop from a single cell, the zygote, which is made up of an egg and a sperm head, both of which contain a haploid genome. At the time of fertilization, the DNA of both egg and sperm is packaged into chromatin, and each has its own epigenetic (DNA methylation and histone modification) ‘state’ related to the previous functional requirements of these cell types. Once fertilization occurs, it is necessary that these epigenetic marks undergo extensive reprogramming for a complex multicellular organism to develop and differentiate. A similar period of extensive reprogramming of the epigenome has been shown to occur in the primordial germ cells during the development of the mature gametes. Some genes, called imprinted genes, are known to escape the epigenetic reprogramming in the early embryo and maintain the epigenetic state established in the gametes of the parents. This observation has supported the idea that perhaps some loci can escape both the reprogramming that occurs during early development and that which occurs during the development of mature gametes, thereby enabling Lamarckian inheritance. The evidence that this happens is scant, but has attracted much attention.

In a recent study published in *Genome Biology*, Iqbal and colleagues [1] have investigated the epigenetic changes that occur to the genome in response to endocrine disruptors and find that these changes are corrected by germline reprogramming events in the next generation.

## The Lamarckian revival

Lamarckian inheritance is the theory that an organism can pass on phenotypes that it acquired during its lifetime to its offspring. This theory was first postulated at the start of the 19th century, but by the end of that century the model of genetic inheritance, from Darwin and Mendel, became preferred. Over the past decade, a handful of studies carried out in mammals have provided support for the idea that exposure to environmental events can drive phenotypic changes that are inherited for more than one generation, and that this occurs through epigenetic mechanisms. One of the key studies driving recent support for Lamarckian inheritance [2] reported that the exposure of pregnant female rats to the endocrine disruptor vincozolin affected male fertility in subsequent generations and that these effects were associated with epigenetic changes in the germ line.

A few independent studies of a range of environmental exposures, such as to bisphenol A, also reported that the resulting phenotype was associated with epigenetic changes in the next generation [3]. Evidence that such effects last for more than one generation has been inconclusive (reviewed in [4-6]). In some instances, effects have been reported following exposure of the male parent to a ‘stress’ [7,8]. For example, offspring of male mice that had been fed a low-protein diet showed changes in the expression of genes involved in cholesterol biosynthesis and changes in DNA methylation [8]. In parallel studies of human populations, it has been suggested that abnormal phenotypes caused by stressors, such as low nutrient intake, might be passed on for many generations through epigenetic marks on the gametes of one parent [9-11]. From these studies, the hypothesis has emerged that environmental ‘stress’ results in epigenetic changes at some loci in the genome and that these can escape the epigenetic reprogramming that normally occurs between generations, the end result being a Lamarckian form of inheritance.

Although the topic is certainly controversial and stimulates robust discussions in informal settings, studies that refute the idea are mainly absent from the literature. It is very difficult to publish negative results, no matter how important those negative results might be. The end result is that the published studies supporting Lamarckian inheritance seem to be uncontested to those outside the field. As a result, many people who are unfamiliar with the molecular sciences and who may be less able to critically assess the evidence are getting an incomplete story.

## Epigenetic consequences of exposure to endocrine disruptors

Iqbal and colleagues [1] specifically set out to identify any transcriptional changes or DNA methylation changes that could explain the reported transgenerational effects of *in utero* exposure to endocrine disruptors in mice. They hypothesized that for epigenetic changes to be passed to a grandchild (G2), the endocrine disruptors must have their effects on the epigenome of the germ cells in the first generation (G1) while *in utero*. In other words, the effects of exposure must occur while the developing G1 embryo is in the uterus of the G0 female. In addition, to affect further generations, such as the great-grandchild, the modifications must persist in the germ cells of the G2 grandchild, who was not exposed to endocrine disruptors at any point during development.

The authors [1] used expression arrays on mRNA purified from germ cells to study global expression patterns, and several methods to study DNA methylation at imprinted loci, at CpG islands and at promoters. They detected changes in transcription and methylation in the G1 germline immediately after exposure to the chemicals. Contrary to previous hypotheses, they found that these epigenetic changes did not persist into the G2 germline. In addition, they looked for effects of these chemicals on the establishment of genomic imprints but found no persistent abnormalities in DNA methylation at the differentially methylated regions of imprinted genes. Previous studies [12] have reported that the process of genomic imprinting is perturbed by *in utero* exposure to endocrine disruptors in further generations. Of course, it is impossible to completely rule out any vestigial epigenetic marks or any vestigial effects on the mRNA population, but Iqbal and colleagues [1] have carried out a detailed and extensive study. They conclude that although the endocrine disruptors exert direct epigenetic effects in the exposed fetal germ cells, these are corrected by reprogramming events in the next generation.

This paper [1] provides a citable reference for the ‘doubters’ of Lamarckian inheritance in mammals and, as such, is a valuable contribution to this ongoing debate.
